# How larvae feel the world around them

**DOI:** 10.7554/eLife.96708

**Published:** 2024-03-08

**Authors:** Jimena Berni

**Affiliations:** 1 Department of Neuroscience, Brighton and Sussex Medical School, University of Sussex Brighton United Kingdom

**Keywords:** perception, sensilla, mechanosensation, *Drosophila*, information processing, larvae, *D. melanogaster*

## Abstract

A complete map of the external sense organs shows how fruit fly larvae detect different aspects of their environment.

**Related research article** Richter V, Rist A, Kislinger G, Laumann M, Schoofs A, Miroschnikow A, Pankratz M, Cardona A, Thum AS. 2024. Morphology and ultrastructure of external sense organs of *Drosophila* larvae. *eLife* **12**:RP91155. doi: 10.7554/eLife.91155.

All animals are exposed to a changing environment. In order to adapt and survive, they need to gather information about their surroundings and choose how best to respond to each condition. For instance, the small larvae of the fruit fly *Drosophila melangoster* face several decisions as they crawl and dig through the decaying vegetable matter they inhabit: how much heat or fermented alcohol should they tolerate? Which chemical trace should they follow? And should they stay or escape if they sense something (which might be a predator) contact their body?

The larvae perceive the world around them through a complex array of external sense organs that each receive particular environmental cues, such as olfactory, gustatory, temperature or mechanosensory signals (Karkali and Martin-Blanco, 2017; Gomez-Marin et al., 2011; Apostolopoulou et al., 2015; Klein et al., 2015). The sensory organs then relay this information to other cells in the nervous system, which trigger the fly to enact the most appropriate behaviour and physiological response.

Knowing the structure and location of external sense organs can provide new insights into how an animal is able to perceive changes in their environment, including identifying the neural pathways that integrate this sensory information and control how the animal will respond. It also offers fundamental information about which features an animal is interpreting in their surroundings.

In insects, knowing the anatomy of an external sensor is also particularly informative as their bodies are covered by an impermeable and relatively rigid exoskeleton called the cuticle. Most sense organs contain one or more hair-like protrusions, known as sensilla, which have specific characteristics that make them good at detecting certain environmental cues (Chapman, 2013). For instance, the sensilla responsible for mechanosensation are attached to a flexible joint which allows them to perceive the direction and force of a mechanical stimuli. Meanwhile the sensilla for olfaction have many little gaps within the cuticle so that volatile smell molecules can infiltrate and bind to the sensor. Now, in eLife, Andreas Thum and co-workers – including Vincent Richter as first author – report the first complete anatomical description of all the external sense organs of fruit fly larvae (Richter et al., 2024).

The team (who are based at the University of Konstanz, Leipzig University, University of Cambridge, University of Bonn, and German Centre for Integrative Biodiversity Research) imaged the body of the larvae using three-dimensional electron microscopy. From these images, they were able to determine the external structure of each sense organ by evaluating the anatomy of the sensilla, as well as their associated sensory and accessory (support) cells. Three types of sensilla were identified on the thorax and abdomen of the larvae – named hair, papilla and knob – which either sat alone or clustered together into small groups forming the sense organs (Figure 1). Most of these sensilla displayed structural properties commonly found in mechanosensory cells that perceive information related to pressure, vibration and movement (Karkali and Martin-Blanco, 2017).

**Figure 1.**
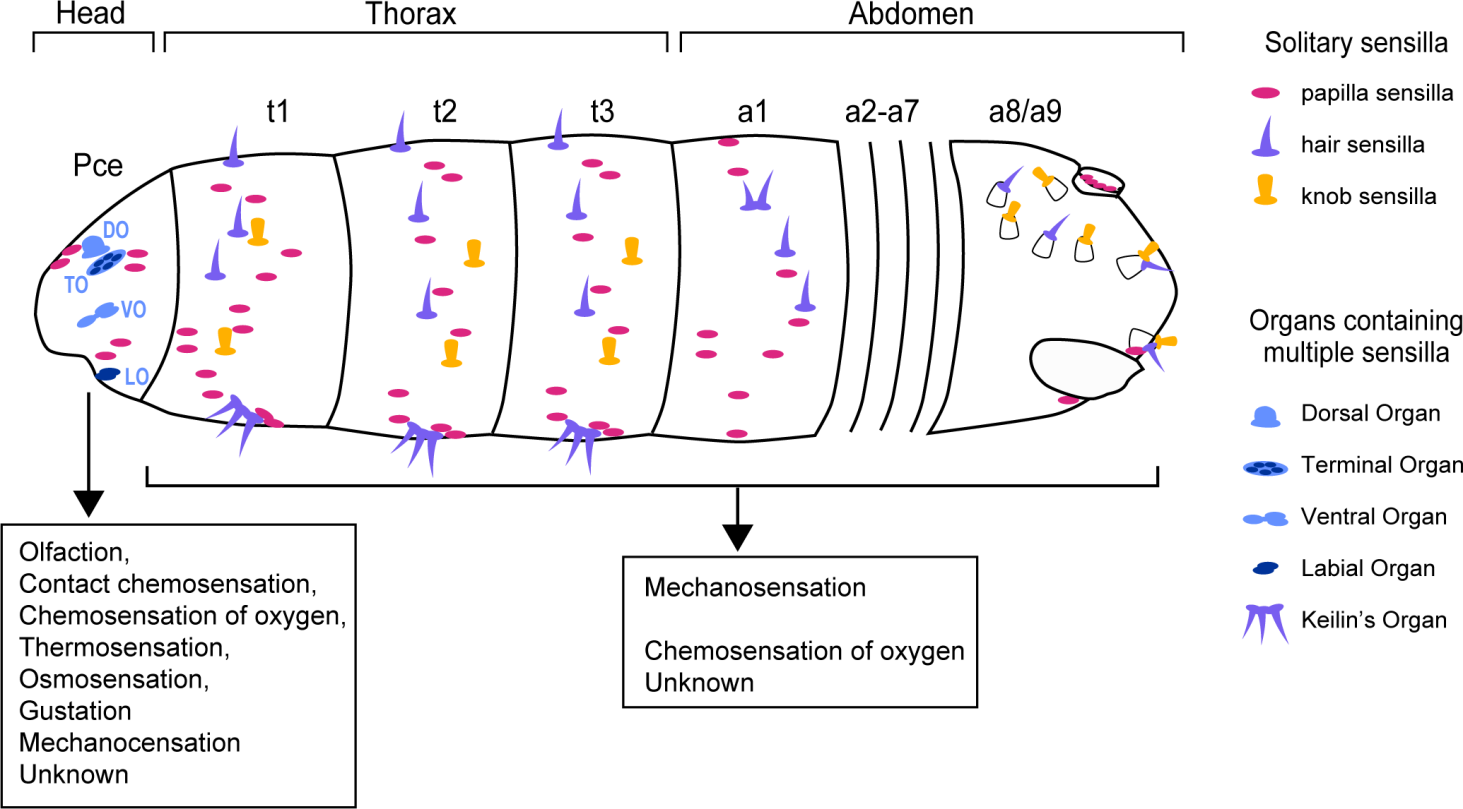
Anatomy and function of the external sense organs. Distributed across the surface of fruit fly larvae are numerous external sense organs that detect particular environmental cues. Along the thorax and abdomen are protrusions, known as sensilla, which were grouped into three categories – papilla (pink), hair (purple), and knob (yellow) – based on how well their shape related to a previous classification (Dambly-Chaudière and Ghysen, 1986). The sensilla are either solitary or grouped together into structures called organs (light and dark blue). Most sensilla in the thorax and abdomen are devoted to mechanosensation, with a small proportion involved in chemosensation or an unknown role. The head of the larvae, known as the pseudocephalon (Pce), contains four sense organs which each contain multiple sensilla: the dorsal organ (DO), terminal organ (TO), ventral organ (VO) and labial organ (LO). Each of these organs detects a specific set of environmental cues, such as contact chemosensation, thermosensation, osmosensation, gustation and mechanosensation.

Figure credit: Adapted from Figure 1B in Richter et al., 2024.

The head of the larvae (also known as the pseudocephalon) contained the highest number and most diverse range of sensilla. Most of these resided in sense organs which each had their own distinct characteristics (Figure 1). This included certain structures and cells that are known to be required for detecting particular environmental cues, including chemicals, temperature, taste and smell among others (Rist and Thum, 2017; Couto et al., 2005, Kwon et al., 2007; Klein et al., 2015). For instance, in the sense organs hypothesized to detect changes in temperature, the sensilla typically had two staked neurons, the lower one forming extensive lamellation, similar to the one seen in the dorsal organ.

The findings of Richter et al. provide new insights in to how fruit fly larvae behave in their natural habitat. In the future, this approach could be applied to other ‘maggot’ species living in different environments to compare how their sensory system influences their behaviour. Notably, Richter et al. also found some sense organs contained multiple dendrites that sense different types of external stimuli. This suggests that there could be cross talk between sensory inputs, and genetic tools available in the fruit fly could be employed to explore this possibility.

There is no doubt that the exquisite description of the external sense organs by Richter et al. will accelerate our understanding of how environmental cues are perceived and processed to generate an appropriate response. Furthermore, combining this information with the tools available to label and manipulate the activity of sensory organs, as well as their partner neurons (Winding et al., 2023), offers a unique opportunity to investigate how animals perceive the world around them.
